# A Membrane Fusion Protein αSNAP Is a Novel Regulator of Epithelial Apical Junctions

**DOI:** 10.1371/journal.pone.0034320

**Published:** 2012-04-02

**Authors:** Nayden G. Naydenov, Bryan Brown, Gianni Harris, Michael R. Dohn, Victor M. Morales, Somesh Baranwal, Albert B. Reynolds, Andrei I. Ivanov

**Affiliations:** 1 Department of Medicine, University of Rochester, Rochester, New York, United States of America; 2 Department of Cancer Biology, Vanderbilt University, Nashville, Tennessee, United States of America; University of Washington, United States of America

## Abstract

Tight junctions (TJs) and adherens junctions (AJs) are key determinants of the structure and permeability of epithelial barriers. Although exocytic delivery to the cell surface is crucial for junctional assembly, little is known about the mechanisms controlling TJ and AJ exocytosis. This study was aimed at investigating whether a key mediator of exocytosis, soluble N-ethylmaleimide sensitive factor (NSF) attachment protein alpha (αSNAP), regulates epithelial junctions. αSNAP was enriched at apical junctions in SK-CO15 and T84 colonic epithelial cells and in normal human intestinal mucosa. siRNA-mediated knockdown of αSNAP inhibited AJ/TJ assembly and establishment of the paracellular barrier in SK-CO15 cells, which was accompanied by a significant down-regulation of p120-catenin and E-cadherin expression. A selective depletion of p120 catenin effectively disrupted AJ and TJ structure and compromised the epithelial barrier. However, overexpression of p120 catenin did not rescue the defects of junctional structure and permeability caused by αSNAP knockdown thereby suggesting the involvement of additional mechanisms. Such mechanisms did not depend on NSF functions or induction of cell death, but were associated with disruption of the Golgi complex and down-regulation of a Golgi-associated guanidine nucleotide exchange factor, GBF1. These findings suggest novel roles for αSNAP in promoting the formation of epithelial AJs and TJs by controlling Golgi-dependent expression and trafficking of junctional proteins.

## Introduction

Intercellular junctions are the most characteristic morphological features of differentiated epithelia. These plasma membrane structures mediate physical interactions between adjacent epithelial cells thereby ensuring the integrity of epithelial layers and creating a barrier to free paracellular passage of different substances. Furthermore, junctions are essential for establishing the apico-basal cell polarity that determines absorptive or secretion phenotypes of epithelial cells [1], [2]. Several types of junctions have been identified in mammalian epithelia including tight junctions (TJs), adherens junctions (AJs), desmosomes, and gap junctions [1], [2], [3]. The most apically located TJs and subjacent AJs are considered key regulators of the paracellular barrier and epithelial cell polarity.

Adhesive properties of epithelial junctions are determined by specialized integral membrane proteins such as the AJ constituent E-cadherin, or the TJ components claudins, occludin, and junctional adhesion molecule (JAM)-A [4], [5], [6], [7], [8]. By interacting with their partners on opposing plasma membrane, these integral proteins mediate cell-cell attachment and formation of the paracellular barrier. On the cytosolic face of the plasma membrane, the transmembrane junctional constituents interact with scaffolding, cytoskeletal and signaling proteins to form cytosolic plaques of AJs and TJs [4], [5], [6], [7], [8]. Components of the AJ cytosolic plaque, α-, β- and p120 catenins, and the TJ plaque proteins, zonula occludens (ZO)1–3 enhance adhesive properties of epithelial junctions and regulate AJ/TJ biogenesis [5], [6], [7], [8], [9].

The current paradigm considers AJs and TJs as highly dynamic structures that undergo constant remodeling (disassembly and reassembly) [10], [11], [12], [13], [14]. Such junctional plasticity is essential for the reorganizations of epithelial layers during normal tissue morphogenesis, but can also lead to epithelial barrier disruption in several diseases [10], [13], [15], [16]. The body of evidence suggests that the remodeling of AJs and TJs is regulated by vesicle trafficking, where disassembly and reassembly steps are mediated by the endocytosis and exocytosis of junctional proteins respectively [15], [17], [18]. Internalization of AJ/TJ proteins is well documented in epithelial cell monolayers challenged with various pathogenic stimuli and has been summarized in several recent reviews [10], [13], [16], [18], [19]. By contrast, the functional roles and mechanisms of AJ/TJ exocytosis remain poorly understood, although this process has been implicated in the formation and maintenance of epithelial barriers. For example, a steady-state exocytosis of E-cadherin and occludin has been detected in confluent epithelial monolayers with functional junctions [20], [21], [22], [23] and disruption of exocytosis was shown to attenuate reformation of AJs and TJs [20], [24], [25], [26], [27]. One can therefore suggest that inhibition of junctional protein exocytosis may represent an important mechanism, by which various pathogenic stimuli disrupt the integrity of epithelial barriers.

Exocytosis is a directional transport of vesicles from the cytoplasm to the plasma membrane [28]. This multistep process involves tethering, docking and fusion of vesicles with the target membrane and is controlled by a variety of accessory and signaling proteins [28], [29], [30], [31]. The ultimate fusion of two phospholipid membranes is a critical event of exocytosis that is mediated by the SNARE (Soluble N-ethylmaleimide-sensitive factor Associated Receptor) multiprotein complex [32], [33], [34], [35]. Different components of the SNARE machinery are located on both the vesicle and the target membranes; their interactions bring these membranes into close opposition to create a fusion pore [32], [33], [34], [35]. SNARE-mediated membrane fusion appears to be important for assembly of epithelial AJs and TJs [27], [36], although the molecular details and regulatory mechanism of this process are unknown.

To support a continuous exocytosis, mature SNARE complexes must be constantly disassembled and reused for new membrane fusion events. Such SNARE disassembly and recycling is mediated by N-ethylmaleimide Sensitive Factor (NSF) and its adaptors, soluble NSF-attachment proteins (SNAPs) [32], [33], [34], [37], [38]. Mammalian cells express α, β and γSNAPs among which the α and γ isoforms are ubiquitous, while βSNAP is predominantly expressed in neural cells [39]. αSNAP acts by recruiting NSF to the SNARE complex, stimulating NSF activity and transducing conformational changes from NSF that lead to disassembly of the SNARE cylinder [40], [41]. Additionally, αSNAP is known to interact with other proteins; however, the functional roles of such interactions remain unknown [39], [42], [43], [44], [45]. A handful of previous studies implicated αSNAP in the regulation of cell-cell adhesions. For example, siRNA mediated knockdown of this protein in vascular endothelium was shown to disrupt VE-cadherin-based AJs [46]. Furthermore, an αSNAP mutation that dramatically decreases its protein expression in *hyh* mice resulted in distorted apico-basal cell polarity in the neuroendocrine epithelium [47]. Still, the exact roles and mechanisms of αSNAP-dependent regulation of epithelial junctions are unknown.

In the present study we examined the involvement of αSNAP in regulating structure and barrier properties of apical junctions in cultured human intestinal epithelial cells. We report here that αSNAP plays key roles in controlling the structural integrity of AJs and TJs and the development of the paracellular barrier. Molecular mechanisms underlying αSNAP-dependent regulation of epithelial junctions may involve control of the Golgi integrity and expression of AJ proteins.

## Results

### Depletion of αSNAP prevents development of the paracellular barrier and assembly of apical junctions in intestinal epithelial cells

**Figure 1:**
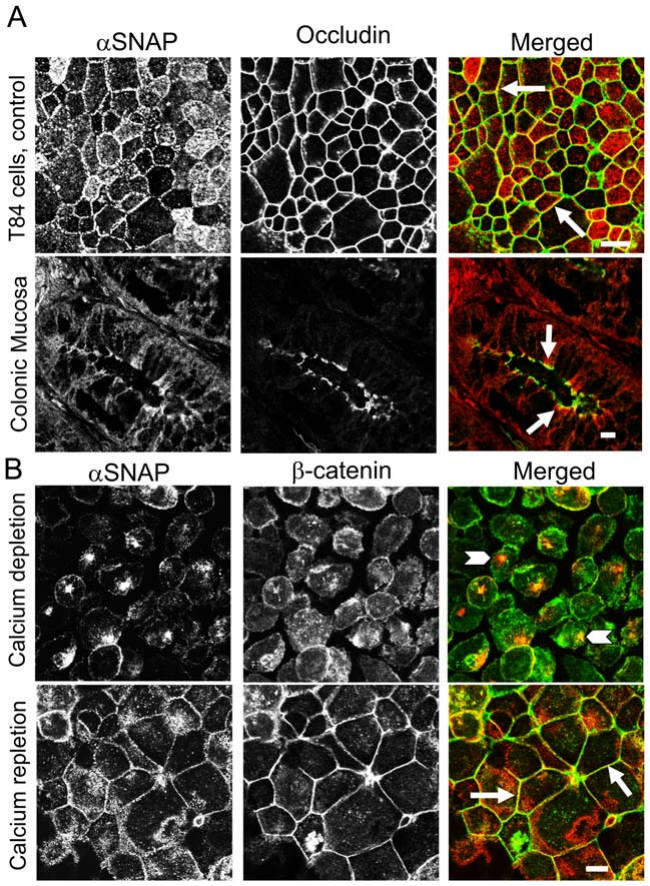
αSNAP is enriched at epithelial junctions *in vitro* and *in vivo.* (**A**) Dual immunofluorescence labeling and confocal microscopy show colocalization (arrows) of αSNAP (red) and tight junction protein occludin (green) in confluent T84 cell monolayers and normal human colonic mucosa. (**B**) Immunofluorescence images show predominantly-perinuclear localization of αSNAP in calcium-depleted SK-CO15 cells (arrowheads) and its rapid translocation to newly-formed AJs in calcium-repleted cells (arrows). Scale bar, 10 µM.

In order to mediate delivery of AJ/TJ proteins to apical junctions αSNAP must be enriched at the areas of cell-cell contact. We analyzed αSNAP localization in polarized T84 colonic epithelial cell monolayers by dual immunolabeling and confocal microscopy, which showed a significant colocalization of αSNAP with occludin at mature TJs (Figure 1A). This *in vitro* observation was reinforced by immunolabeling of normal human intestinal mucosa that also revealed enrichment of αSNAP at apical junctions and its colocalization with occludin in crypt epithelium (Figure 1A, arrows). We next used a calcium switch model to investigate if αSNAP is involved in early stages of junction assembly. In contact-naïve, calcium-depleted SK-CO15 colonic epithelial cells, β-catenin demonstrated a diffuse cytosolic labeling, whereas αSNAP accumulated in the perinuclear region (Figure 1B, arrowheads). Re-addition of extracellular calcium (calcium repletion) triggered rapid (within 1 h) translocation of β-catenin to newly forming AJ-like junctions (Figure 1B). Similarly, αSNAP showed early relocalization to nascent cell-cell contacts during calcium repletion (Figure 1B, arrows). Together, these data indicate a close association of αSNAP with epithelial junctions *in vitro* and *in vivo*.

**Figure 2:**
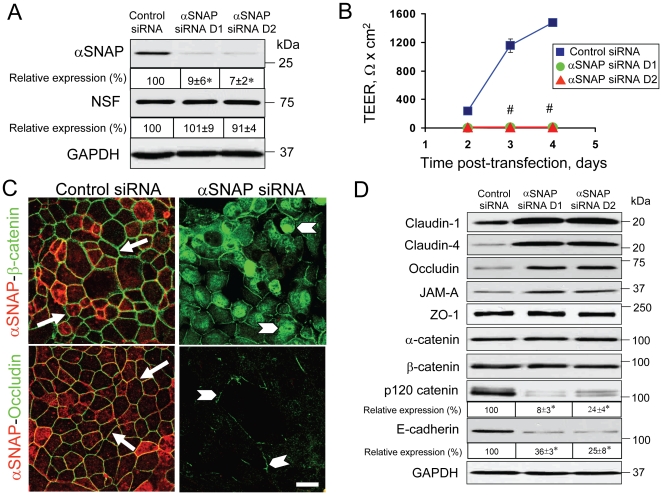
siRNA-mediated depletion of αSNAP prevents formation of the paracellular barrier and AJ/TJ assembly, and selectively downregulates p120 catenin and E-cadherin expression. (**A**) Immunoblotting analysis shows that two different αSNAP-specific siRNA duplexes (D1 & D2) dramatically decrease its protein expression in SK-CO15 cells on day 3 post-transfection. (**B**) TEER measurements demonstrate that αSNAP-depleted SK-CO15 cell monolayers fail to develop the paracellular barrier. (**C**) Immunofluorescence labeling shows formation of normal β-catenin-based AJs and occludin-based TJs (arrows) in control SK-CO15 cell monolayers on day 4 post-transfection. By contrast, αSNAP-depleted cells have significant intracellular accumulation of β-catenin and fragmented TJ strands (arrowheads). (**D**) Immunoblotting analysis shows that selective αSNAP depletion increases the level of TJ proteins but dramatically downregulates p120 catenin and E-cadherin expression in SK-CO15 cells on day 4 post-transfection; *p<0.05, ^#^p<0.01 compared to the control siRNA-treated group (n = 3). Scale bar, 20 µM.

To examine the roles that αSNAP plays in regulation of AJs and TJs we used small interfering (si) RNA to downregulate this protein in SK-CO15 cells. We found that two different αSNAP-specific siRNA duplexes (D1 and D2) decreased its protein expression by up to 93% on day 3 (Figure 2A) and day 4 (data not shown) post-transfection, without affecting the protein level of NSF. Remarkably, such αSNAP depletion completely prevented establishment of the paracellular barrier. SK-CO15 cells plated on membrane filters and transfected with control siRNA steadily developed a transepithelial electrical resistance (TEER) that reached approximately 1500 Ω×cm^2^ by day 4 post-transfection (Figure 2B). In contrast, the TEER values of αSNAP-depleted cell monolayers did not exceed 10 Ω×cm^2^ under these experimental conditions (Figure 2B). Furthermore, immunofluorescence labeling and confocal microscopy revealed the typical ‘chicken wire’ staining pattern of β-catenin and occludin in the control SK-CO15 cells on day 4 post-transfection (Figure 2C, arrows), which is indicative of well-formed AJs and TJs. In contrast, αSNAP-depleted cells were characterized by predominantly intracellularly-located β-catenin and short disconnected areas of occludin labeling at cell-cell contacts (Figure 2C, arrowheads). αSNAP knockdown also dramatically interrupted junctional recruitment of claudin-4, ZO-1 and α-catenin (data not shown) thereby indicating global disruption of TJ and AJ structure.

We next asked whether the observed cell-cell adhesion defects in αSNAP-depleted cells were due to altered expression of AJ/TJ proteins. Downregulation of αSNAP with two different siRNA duplexes selectively decreased the levels of p120 catenin and E-cadherin by up to 92% and 75% respectively on day 4 post-transfection (Figure 2D). Loss of p120 catenin protein was already obvious on day 2 of the αSNAP knockdown (data not shown), whereas the decrease in E-cadherin level occurred later, on day 4 post-transfection. In contrast, expression of TJ proteins such as claudins-1 and 4, occludin and JAM-A, was significantly increased by αSNAP depletion (Figure 2D). All together, our data indicate that the loss of αSNAP triggers profound disruption of epithelial apical junctions and downregulation of key AJ proteins.

### Effects of αSNAP depletion on the integrity of epithelial junctions and the paracellular barrier are independent of apoptosis

**Figure 3:**
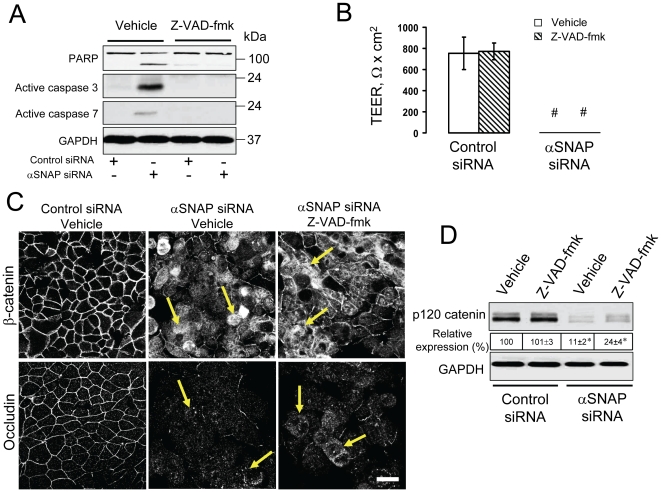
Disruption of apical junctions and downregulation of p120 catenin expression in αSNAP-depleted cells are not mediated by apoptosis. (**A**) Immunoblotting analysis of cleaved PARP and active caspases 3 & 7 shows that αSNAP depletion causes significant cell apoptosis in SK-CO15 cells on day 4 post-transfection, which is prevented by inhibition of caspases with Z-VAD-fmk (50 µM). (**B–D**) To examine the role of apoptosis in junction disassembly, SK-CO15 cells were transfected with either control or αSNAP-specific siRNA (duplex 1), and one day later, were exposed to either vehicle or Z-VAD-fmk (50 µM) for 72 h. Inhibition of apoptosis does not prevent disruption of the paracellular barrier (**B**), disruption of AJs and TJs (**C**, arrows) or down-regulation of p120 catenin expression (**D**) in αSNAP-depleted epithelial cells; *p<0.05, ^#^p<0.01 compared to the control siRNA-treated group (n = 3). Scale bar, 20 µm.

While examining the effects of αSNAP-knockdown on the integrity of the epithelial barrier, we noticed another phenotype involving cell rounding and detachment, which could be indicative of cell death (data not shown). Therefore, we examined activation of the most common cell death pathway, apoptosis, in αSNAP-depleted SK-CO15 cells. We observed significant induction of apoptosis, as characterized by PARP cleavage, caspases 3 and 7 activation (Figure 3A) and an increase in cell labeling with FITC-Annexin V (data not shown). This apoptosis induction was prevented by the cell-permeable pan-caspase inhibitor Z-VAD-fmk (Figure 3A, and data not shown). Since apoptosis is known to disrupt epithelial barriers and induce degradation of some junctional proteins, we asked if this cell death mechanism was responsible for AJ/TJ disassembly and downregulation of p120 catenin caused by αSNAP depletion. SK-CO15 cells were transfected with either control or αSNAP-specific siRNA (duplex 1), and one day later were treated for 72 h with either vehicle (DMSO) or Z-VAD-fmk (50 µM). Caspase inhibition failed to prevent disruption of the paracellular barrier (Figure 3B), AJ and TJ disassembly (Figure 3C), and did not restore normal levels of p120 catenin (Figure 3D) in αSNAP-depleted cells. In some experimental systems, caspase inhibitors can switch the cell death program from apoptosis to necroptosis. To exclude this possibility, we performed additional experiments involving a dual inhibition of apoptosis and necroptosis by a combination of Z-VAD-fmk and necrostatin (Nst)-1. Such co-inhibition of two major cell death pathways failed to prevent junctional disassembly in αSNAP-depleted SK-CO15 cells (Figure S1, arrowheads).

**Figure 4:**
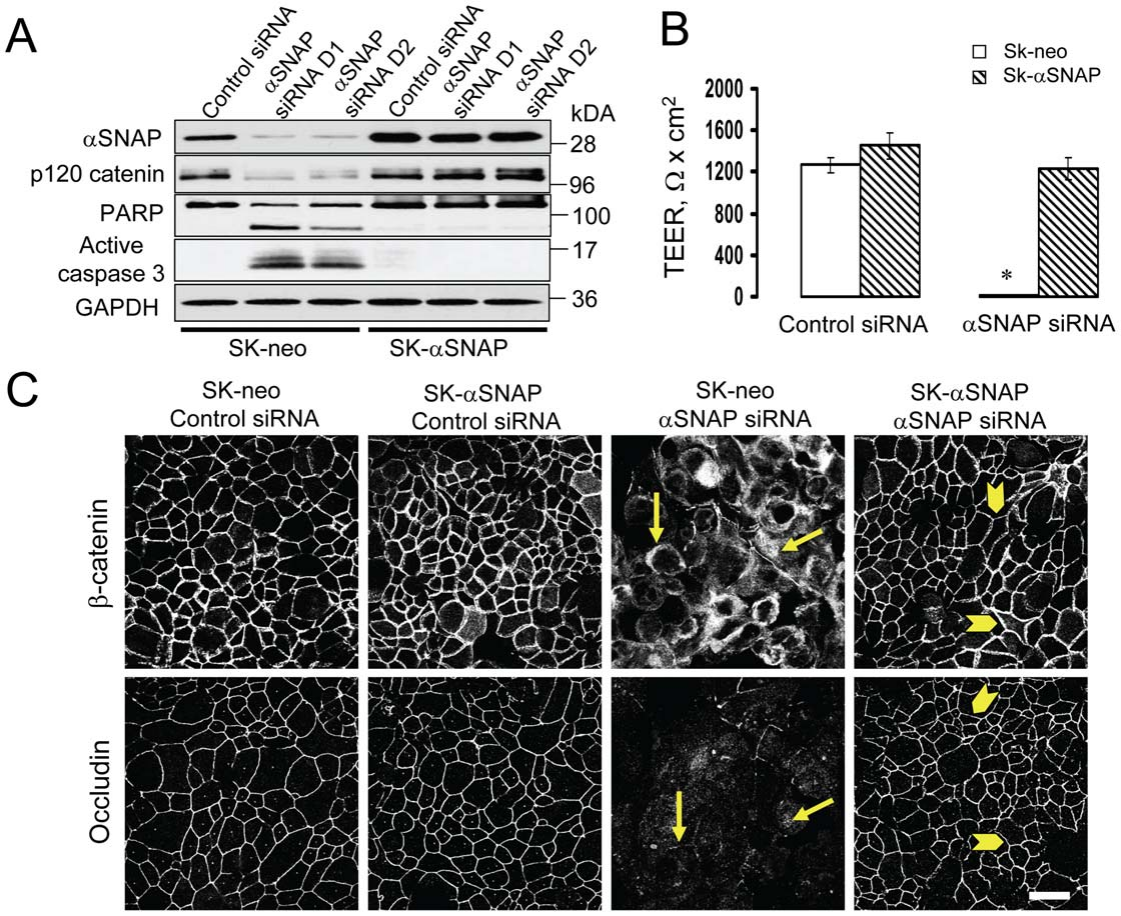
Junctional disassembly and apoptosis in αSNAP-depleted cells can be rescued by expression of siRNA-resistant bovine αSNAP. (**A**) Immunoblotting analysis shows preserved αSNAP level in SK-CO15 cells with stable expression of bovine αSNAP (SK-αSNAP) after siRNA depletion of endogenous human protein. Comparing to the control cells (SK-neo), such bovine αSNAP overexpression completely prevents induction of apoptosis (**A**), disruption of the paracellular barrier (**B**) and AJ/TJ disassembly (**C, arrowheads**) caused by loss of endogenous αSNAP; ^#^p<0.01 compared to the control siRNA-treated group (n = 3). Scale bar, 20 µm.

To ensure that the observed cellular changes caused by αSNAP knockdown did not represent off-target effects of siRNA expression, we performed rescue experiments involving overexpression of bovine αSNAP lacking complementation for human siRNA sequences. Remarkably, expression of this siRNA-resistant protein completely reversed the decrease in paracellular permeability, AJ/TJ disassembly and loss of p120 catenin expression caused by αSNAP knockdown (Figure 4A–C). It also prevented epithelial cell apoptosis (Figure 4A). Together, these experiments strongly suggest that disruption of apical junctions and induction of apoptosis represent two specific and independent functional consequences of αSNAP depletion in human epithelial cells.

### Downregulation of p120 catenin disrupts epithelial AJs and TJs but cannot explain defects of cell-cell adhesions induced by αSNAP knockdown

**Figure 5:**
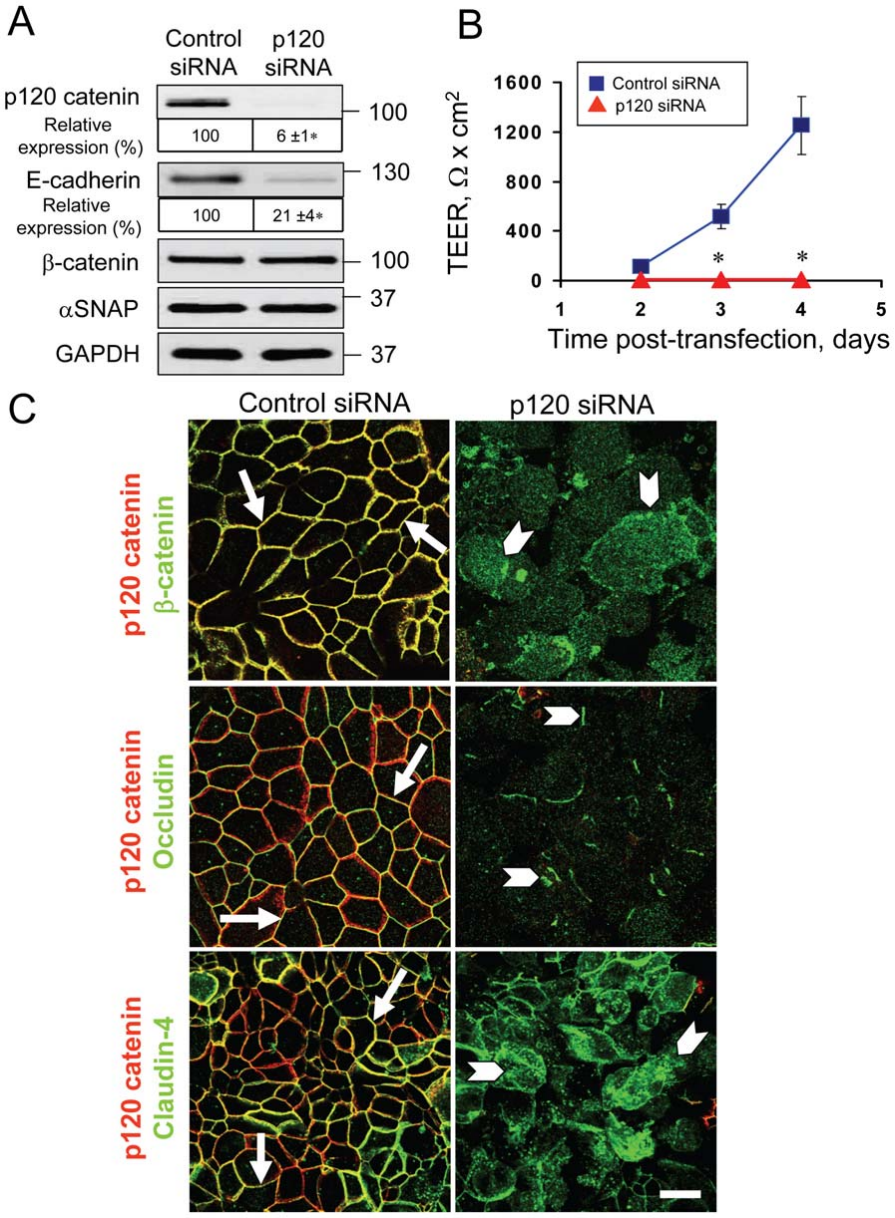
Downregulation of p120 catenin expression phenocopies the effects of αSNAP depletion on epithelial junctions. (**A**) Immunoblotting analysis shows that p120 catenin-specific siRNA dramatically decreases p120-catenin and E-cadherin protein expression in SK-CO15 cells. (**B**) TEER measurements demonstrate that p120 catenin-depleted SK-CO15 cell monolayers do not develop the paracellular barrier on days 2–4 post-transfection. (**C**) Immunofluorescence labeling shows formation of normal β-catenin-based AJs and occludin/claudin-4-based TJs (arrows) in control SK-CO15 cell monolayers. In contrast, p120 catenin-depleted cells display a defective AJ/TJ assembly and intracellular localization of junctional proteins (arrowheads) on day 4 post-transfection; *p<0.01 compared to the control siRNA-treated group (n = 4). Scale bar, 20 µm.

Given the established roles for classical cadherins and p120 catenin in regulation of cell-cell adhesions, one can suggest that downregulation of these proteins plays a role in AJ/TJ disassembly in αSNAP-depleted cells. Previous studies have demonstrated that p120 catenin knockdown decreased cadherin expression and impaired AJ structure in cultured cells and animal tissues [48], [49], [50], [51], [52]. On the other hand, E-cadherin appeared to be dispensable for AJ and TJ integrity [53], [54]. Based on these data, we examined whether the loss of p120 catenin mimics the effects of αSNAP depletion on structure and permeability of epithelial junctions. The p120 catenin-specific siRNA SmartPool decreased this protein level by ∼94% and downregulated E-cadherin expression by ∼80%, but did not substantially affect the amount of αSNAP and β-catenin in SK-CO15 cells (Figure 5A). Interestingly, p120 catenin depletion inhibited development of the paracellular barrier (Figure 5B) and junctional recruitment of β-catenin, occludin (Figure 5C), claudin-4 and ZO-1 (data not shown), thereby phenocopying all the observed effects of αSNAP knockdown on epithelial cell-cell adhesions. Next we asked if overexpression of p120 catenin can prevent AJ/TJ disassembly in αSNAP-depleted SK-CO15 cells. siRNA-mediated knockdown of αSNAP was performed in control cells (SK-neo) and cells stably overexpressing p120 catenin isoforms 1 or 3 (SK-p120-1 and SK-p120-3). Among these stable cell lines, SK-120-3 cells preserved a high level of p120 catenin even after αSNAP depletion (Figure S2A). However, following αSNAP knockdown, p120-overexpressing cells still showed dramatic disruption of the paracellular barrier (Figure S2B) and a similar extent of AJ/TJ disassembly as compared to SK-neo controls (Figure S2C, arrows). These experiments indicate that p120 catenin downregulation alone cannot explain AJ/TJ disassembly in αSNAP-depleted epithelia and, therefore, suggest the existence of alternative mechanisms that cooperate in inducing a dramatic dismantlement of epithelial junctions.

### Effects of αSNAP depletion on epithelial junctions are NSF-independent and associated with Golgi fragmentation

**Figure 6:**
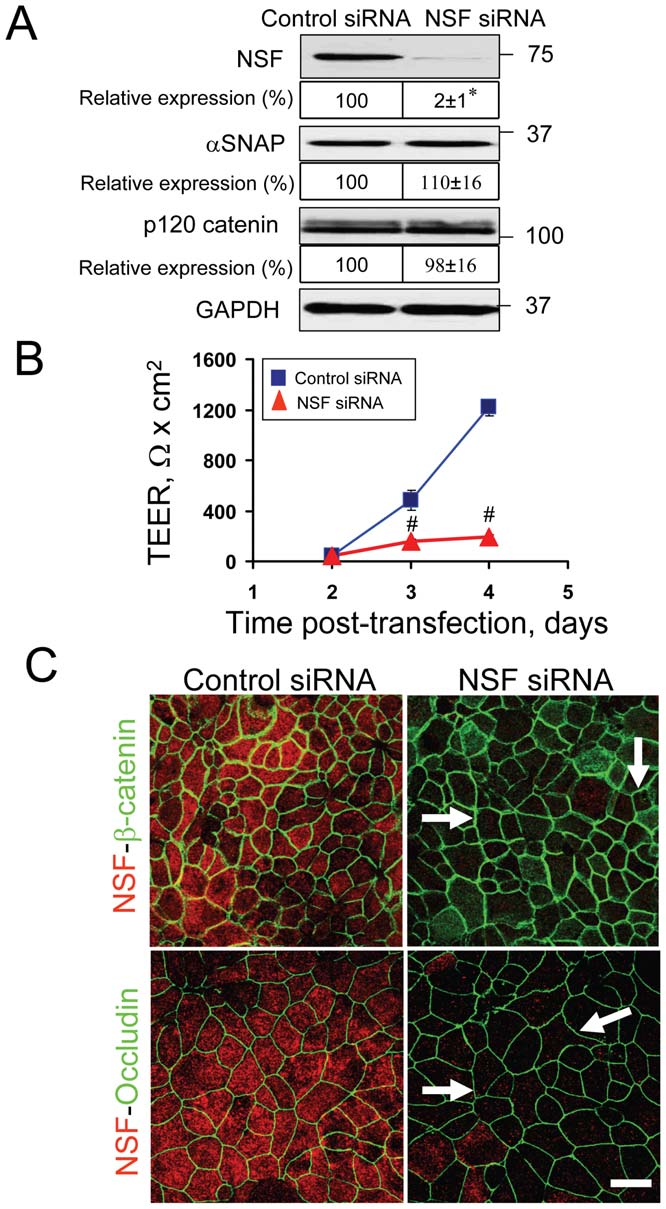
Downregulation of NSF does not phenocopy the major effects of αSNAP knockdown on epithelial junctions. (**A**) Immunoblotting analysis shows that NSF-specific siRNA efficiently decreases expression of the targeted protein without affecting αSNAP and p120 catenin protein levels. (**B**) TEER measurements demonstrate that NSF-depletion significantly attenuates formation of the paracellular barrier in SK-CO15 cell monolayers; *p<0.05 compared to the control siRNA-treated group (n = 3). (**C**) Immunofluorescence labeling shows normal AJ and TJ structure (arrows) in NSF-deficient SK-CO15 cells on day 4 post-transfection; *p<0.05, ^#^p<0.01 compared to the control siRNA-treated group (n = 3). Scale bar, 20 µm.

Since αSNAP regulates protein trafficking to the plasma membrane [28], [37], [39], one can suggest that AJ/TJ disassembly in αSNAP-depleted epithelia reflects inability of vesicles carrying junctional proteins to fuse with the plasma membrane. If this hypothesis is correct, a similar phenotype should be observed after knockdown of NSF, which is the major functional partner of αSNAP in regulating membrane fusion in the exocytic pathway [28], [38]. However, depletion of NSF did not recreate the major effects of αSNAP knockdown on epithelial junctions. First, NSF-depleted SK-CO15 cells demonstrated unaltered expression of p120 catenin (Figure 6A) and E-cadherin (data not shown) as compared to appropriate controls. Second, these cells developed an abnormally-leaky paracellular barrier; however, their TEER values (up to 200 Ω×cm^2^) were still approximately 20 fold higher than those observed in αSNAP-deficient cells (compare Figures 2B and 6B). Third, NSF-depleted cells did not show gross defects in junctional localization of β-catenin and occludin (Figure 6C). Finally, αSNAP and NSF were not essential for each other's stability since downregulation of αSNAP did not significantly alter NSF expression (Figure 2A) and *vice versa* (Figure 6A). Collectively, these results support the conclusion that loss of αSNAP disrupts epithelial junctions via NSF-independent mechanisms.

**Figure 7:**
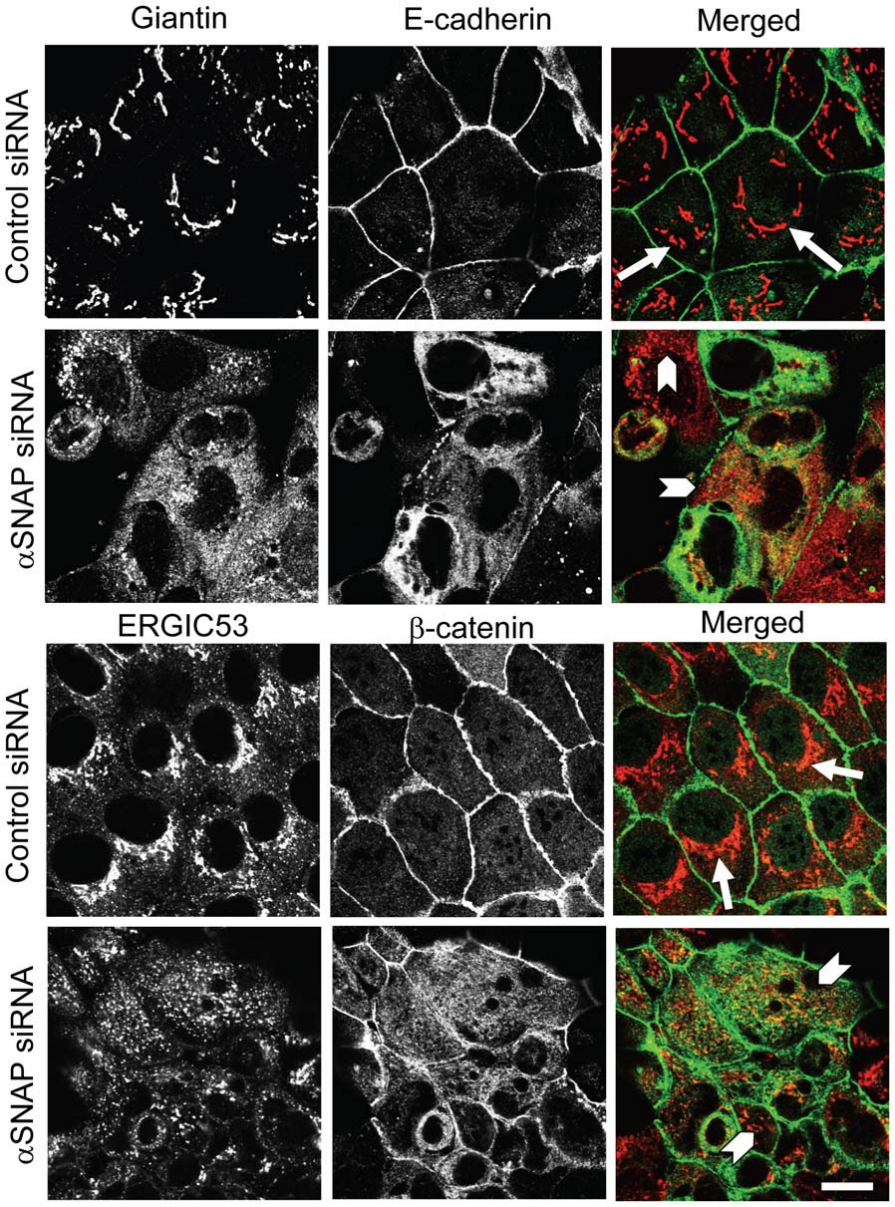
αSNAP depletion induces fragmentation of the Golgi and ERGIC. Control and αSNAP-depleted SK-CO15 cells were immunofluorescence labeled for β-catenin (green) with either Golgi marker GM130, or ERGIC marker ERGIC53 (red) on day 3 post-transfection. Control cells show perinuclear tubular Golgi complexes and well-organized compact ERGIC (arrows). By contrast, αSNAP depletion results in a dramatic dispersion of both perinuclear Golgi and ERGIC structures (arrowheads). Scale bar, 10 µm.

In addition to NSF, αSNAP was shown to interact with several other proteins predominantly localized at the Golgi and the endoplasmic reticulum (ER) [42], [43], [44], [45], [55]. It was also implicated in mediating the reconstituted ER-to-Golgi trafficking [56]. Based on these data, we rationalized that impairment of the Golgi structure and functions may underlie the defects of junctional assembly in αSNAP-depleted epithelial cells. To test this hypothesis we analyzed the effects of αSNAP knockdown on the morphology of the Golgi and the Endoplasmic Reticulum Golgi Intermediate Compartment (ERGIC) by immunolabeling their resident proteins Giantin and ERGIC53 respectively. In control SK-CO15 cells, the Golgi appeared as a set of characteristic tubular structures surrounding the nucleus and the ERGIC had a condensed perinuclear organization (Figure 7, arrows). By contrast, in αSNAP-depleted cells, which can be distinguished by the diffuse intracellular staining of β-catenin, the perinuclear morphology of both Golgi and ERGIC was severely disrupted and Giantin and ERGIC53-containing vesicles were scattered throughout the cell (Figure 7, arrowheads). A quantitative image analysis revealed 91±1% and 5±2% cells with an intact Golgi in the control group and αSNAP-siRNA-treated group respectively (p<0.001; n = 10). Importantly, the observed Golgi and ERGIC fragmentation represented an early effect of αSNAP knockdown already detectable on day 2 post-transfection, and did not reflect late morphological changes observed in apoptotic cells (data not shown). Since dispersion of the Golgi ribbon does not always result in inhibition of its functions [57], we next sought to analyze functional consequences of Golgi fragmentation in αSNAP-depleted cells. We analyzed postranslational modification of β1 integrin, the adhesion protein that requires Golgi-dependent glycosylation for its normal processing and delivery to the cell surface [58], [59]. In control SK-CO15 cells, immunoblotting revealed a characteristic β1 integrin doublet (Figure S3A), in which the upper and the lower bands represented the mature and precursor (non-glycosylated) protein, respectively [58], [59]. Depletion of αSNAP caused a dramatic increase of the intensity of the lower band, thereby indicating accumulation of non-glycosylated β1 integrin (Figure S3A). Consistently, immunofluorescence labeling revealed a prominent cell-cell contact labeling of β1 integrin in control cells (Figure S3B, arrows) that was transformed into intracellular staining following αSNAP knockdown (Figure S3B, arrowheads). These data demonstrate that loss of αSNAP results in the disrupted architecture and impaired functional activity of the Golgi.

**Figure 8:**
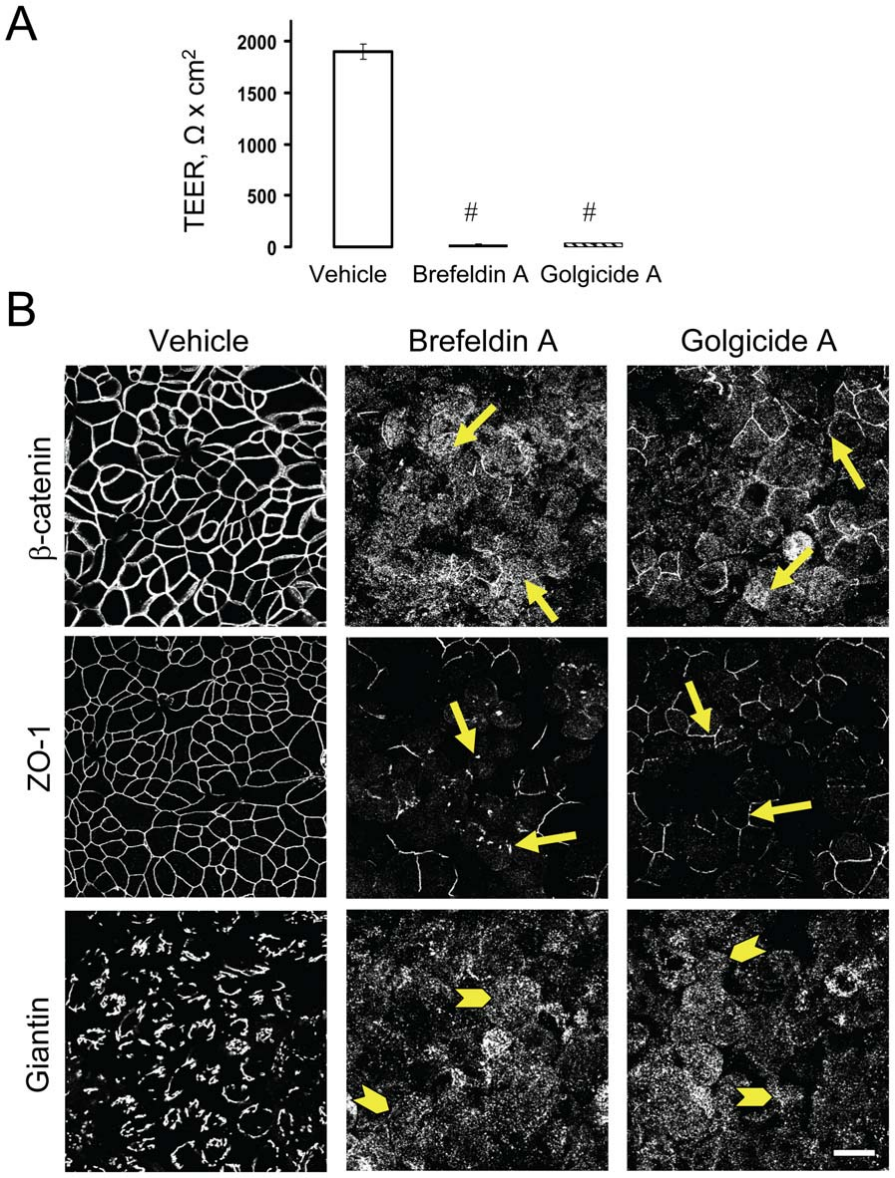
Pharmacological fragmentation of the Golgi induces disruption of the epithelial barrier and disassembly of apical junctions. Immunofluorescence labeling shows that 24 h incubation of SK-CO15 cells with either Brefeldin A (2 µM) or Golgicide A (50 µM) causes a significant increase in paracellular permeability (**A**), dramatic disassembly of AJs and TJs (**B, arrows**), and fragmentation of the Golgi (**B, arrowheads**); ^#^p<0.01 compared to the vehicle-treated group (n = 3). Scale bar, 20 µm.

To establish a possible causal link between Golgi dysfunctions and disassembly of epithelial junctions, we used Brefeldin A (BFA, 2 µM) and Golgicide A (GA, 50 µM), two ‘Golgi toxins’ that are known to impair structure and functions of this organelle [60], [61]. Exposure of confluent SK-CO15 cell monolayers to either BFA or GA for 24 h reproduced all the observed effects of αSNAP depletion, such as disruption of the paracellular barrier (Figure 8A), AJ and TJ disassembly (Figure 8B, arrows), and Golgi fragmentation (Figure 8B, arrowheads). Together, these findings suggest that loss of αSNAP may impair the integrity of epithelial junctions by disrupting Golgi structure and functions, although additional data are required to establish a causal link between these orchestrated events.

### Decreased expression of GBF1 can mediate Golgi fragmentation and junctional disassembly in αSNAP-depleted cells

**Figure 9:**
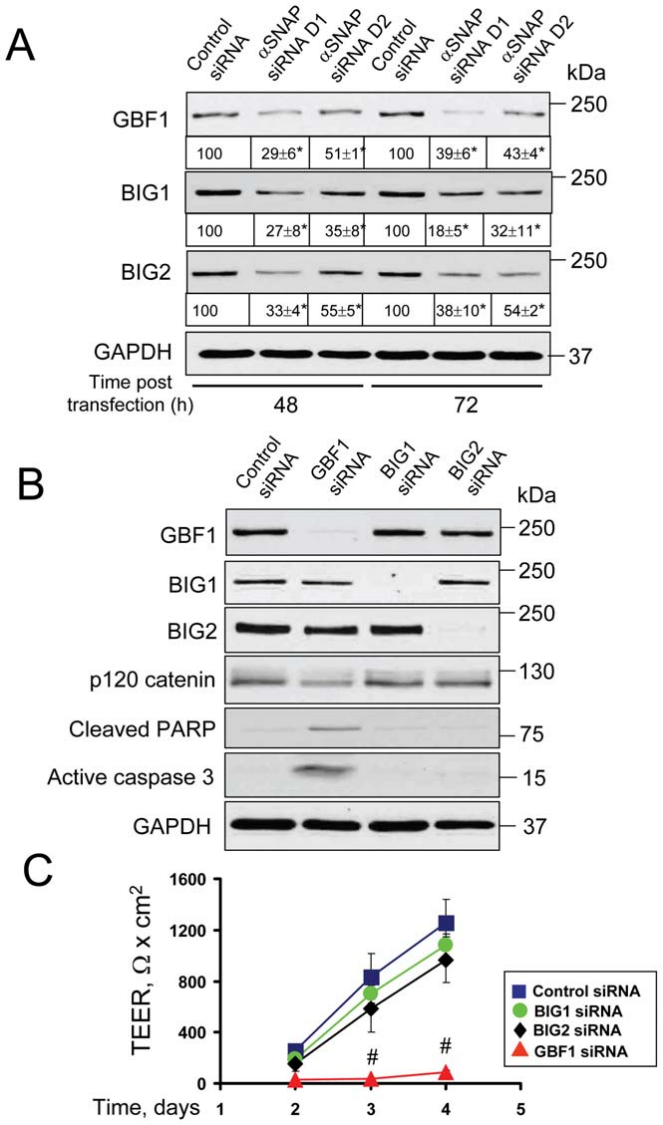
GBF1, but not other Golgi GEFs, regulates barrier functions of the intestinal epithelial junctions. (**A**) Immunoblotting analysis demonstrates that two different αSNAP-specific siRNA duplexes significantly decrease expression of Golgi GEFs, GBF1, BIG1 and BIG2 in SK-CO15 cells on days 2 and 3 post-transfection. (**B,C**) SK-CO15 cells were transfected with either control GBF1, BIG1, or BIG2, siRNAs and examined on day 4 post-transfection. Immunoblotting analysis demonstrates selective down regulation of individual GEFs by their gene-specific siRNAs. Depletion of GBF1, but not BIG1 or BIG2, decreased p120 catenin expression (**B**), dramatically inhibited development of the paracellular barrier (**C**) and induced apoptosis (**B**); *p<0.05, ^#^p<0.01 compared to control siRNA-transfected cells.

**Figure 10:**
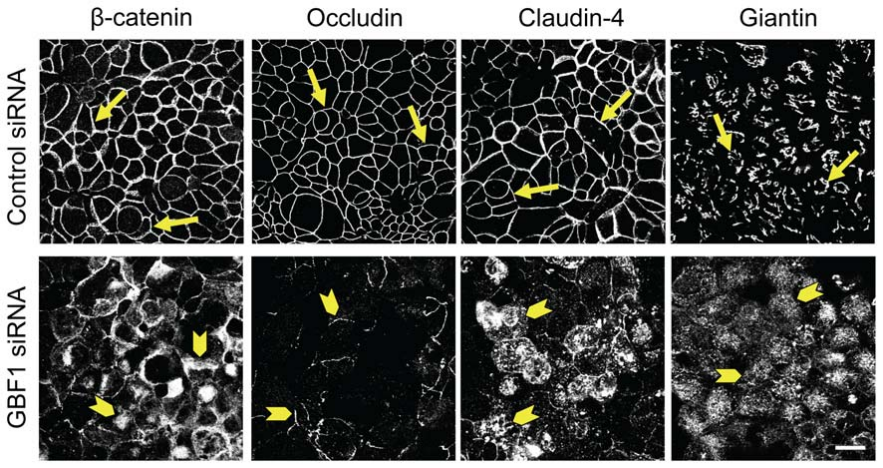
Downregulation of GBF1 expression phenocopies the effects of αSNAP depletion on epithelial junctions. Immunofluorescence labeling shows formation of normal β-catenin-based AJs, occludin/claudin-4-based TJs and well-organized perinuclear Golgi ribbon (arrows) in control SK-CO15 cell monolayers. In contrast, GBF1-depleted cells display a defective AJ/TJ assembly, intracellular localization of junctional proteins and dispersed Golgi (arrowheads) on day 4 post-transfection. Scale bar, 20 µm.

Given the striking similarity between the effects of αSNAP knockdown and ‘Golgi toxins’ on the structure and permeability of epithelial junctions, we sought to explore if these effects are mediated by similar mechanisms. BFA is known to inhibit three different proteins, namely, Golgi brefeldin-sensitive factor 1 (GBF1), BIG1 and BIG2, [62], [63], [64] whereas GA is a selective inhibitor of only GBF1 [61]. All three proteins serve as guanine nucleotide exchange factors (GEFs) for ARF small GTPases that regulate vesicle trafficking between the ER and the Golgi and within the Golgi compartment. Therefore, we asked whether αSNAP knockdown alters expression of the Golgi-associated GEFs. Immunoblotting analysis demonstrated that two different αSNAP-specific siRNAs significantly (by 50–70%) decreased expression of GBF1, BIG1, and BIG2 in SK-CO15 cells at 48 h and 72 h post-transfection (Figure 9A). To examine if diminished expression of these GEFs affects epithelial permeability and organization of AJs and TJs, we individually depleted GBF1, BIG1 and BIG2 using gene-specific siRNAs (Figure 9B). Interestingly, GBF1 knockdown resulted in gross disruption of the epithelial barrier that manifested as a substantially lower TEER (Figure 9C) and loss of normal AJ and TJ labeling (Figure 10, arrowheads). Furthermore, loss of GBF1 decreased expression of p120 catenin and induced apoptosis (Figure 9B), thereby phenocopying all effects observed in αSNAP-depleted epithelium. By contrast, BIG2 knockdown, while slightly decreasing TEER, did not affect junctional architecture and BIG1 depletion did not significantly alter AJ/TJ structure and permeability (Figures 9 & S4). These results highlight the selective role for GBF1 in regulating epithelial junctions and suggest that down-regulation of this Golgi-associated factor may contribute to AJ/TJ disassembly induced by αSNAP knockdown.

## Discussion

### αSNAP regulates AJ and TJ integrity and establishment of the epithelial barrier

αSNAP is a ubiquitously expressed regulator of intracellular vesicle fusion that plays a major role in the disassembly and recycling of various SNARE complexes [37], [39]. Since several SNARE proteins have been previously implicated in the formation of epithelial junctions and the establishment of apico-basal cell polarity [27], [36], it is important to understand if αSNAP is also required for these key events of epithelial differentiation. Our study, for the first time, highlights αSNAP as an indispensible regulator of structure and barrier function of epithelial AJs and TJs. This conclusion is supported by two lines of evidence. One is the enrichment of αSNAP at mature AJs and TJs (Figure 1A) and its cycling between the plasma membrane and cytosolic compartments during formation and disassembly of intercellular contacts (Figure 1B). Interestingly, such junctional accumulation of αSNAP reinforces the old view of TJs as a ‘hot spot’ of vesicle fusion [65], [66]. The other evidence is the loss of barrier function and profound defects in AJ/TJ morphology in αSNAP-depleted epithelial cell monolayers (Figures 2 & 4). This phenotype represents one of the most severe disruptions of apical junctions caused by depletion of a single protein in model mammalian epithelia. Indeed previous studies involving selective downregulation of important structural and regulatory constituents of apical junctions such as occludin, ZO-1, JAM-A, E-cadherin, Scribble, or myosin IIA failed to prevent the ultimate assembly of AJs and TJs and affected only junction remodeling [53], [67], [68], [69], [70]. Nevertheless, our results are consistent with two published reports, which demonstrated disruption of VE-cadherin-based AJs in αSNAP-depleted vascular endothelial cells [46] and described abnormal localization of basolateral plasma membrane proteins in the ventricular neuroepithelium of the mutant *hyh* mice with partial loss of αSNAP expression [47].

### αSNAP controls expression of different junctional proteins

Dramatic effects of αSNAP depletion on the organization of the two major junctional complexes can be determined by multiple mechanisms that disturb several steps of AJ/TJ trafficking and biogenesis. One such mechanism is likely to involve significant imbalance of different junctional components in αSNAP-depleted cells, manifested by expressional downregulation of key AJ constituents and increased levels of TJ proteins (Figure 2D). The latter effect can reflect either compensatory acceleration of synthesis or impaired degradation of TJ proteins and *per se* cannot be responsible for defective cell-cell adhesion. By contrast, the observed loss of p120 catenin and E-cadherin expression is likely to contribute to AJ and TJ disassembly triggered by loss of αSNAP. This notion is supported by the results of p120 catenin knockdown in SK-CO15 cells that mimicked all the effects of αSNAP depletion on epithelial junctions including the lack of paracellular barrier, AJ and TJ disassembly and decreased expression of E-cadherin (Figure 4). Furthermore, our results are in line with previous studies that analyzed effects of genetic depletion of p120 catenin in different cell types *in vitro* and *in vivo* and showed a consistent decrease in protein levels of E-, P-, and N-cadherins and disruption of cell-cell junctions in p120-deficient cells [49], [50], [51], [52], [71]. Surprisingly, overexpression of p120 catenin failed to restore the defects of AJ and TJ structure and barrier function in αSNAP knockdown (Figure S2). However, these negative results do not disprove a possible role for p120 catenin downregulation in αSNAP-dependent junction disassembly, but rather suggest the existence of parallel mechanisms that cannot be overcome by high p120 expression. These mechanisms are not related to induction of cell death in αSNAP-deficient epithelium (Figures 3 & S1), and may involve defective fusion of vesicular AJ/TJ carrier along the exocytic pathways to the plasma membrane or loss of cell-matrix adhesions. The latter idea is supported by the profound defects in β1 integrin processing and localization (Figure S3) and significant detachment of αSNAP-depleted SK-CO15 cells (data not shown) as well as by published studies that implicates cell-matrix adhesions in regulation of epithelial junctions [72], [73].

A surprising finding of this study is that αSNAP-mediated regulation of epithelial apical junctions appears to be independent of its major functional/binding partner, NSF. Indeed, our RNA interference experiments indicate that these proteins do not control each other's stability in intestinal epithelial cells (Figures 2A & 6A). Furthermore, NSF knock-down did not mimic the major effects of αSNAP depletion, such as AJ/TJ disassembly and decreased expression of p120 catenin (Figure 6). These NSF-independent functions of αSNAP, although unusual and poorly-characterized, have been observed previously. For example, transport of resident apical plasma membrane proteins in polarized kidney epithelial cells was shown to be mediated by αSNAP independently of NSF [74]. NSF belongs to a large family of oligomeric ATPases, and other members of this family are known to mediate membrane fusion within different intracellular compartments [55], [75], [76]. It is reasonable to suggest that αSNAP may interact with some NSF homologues to control exocytic trafficking and assembly of epithelial junctions.

### αSNAP regulates integrity of the Golgi complex and expression of Golgi GEFs

Our study suggests that disruption of the Golgi complex may represent a key mechanism that mediates AJ and TJ disassembly in αSNAP-depleted cells. Golgi fragmentation appeared to be one of the most vivid morphological effects of αSNAP knockdown in SK-CO15 cells (Figure 7) and other types of model epithelia (data not shown). Furthermore, dispersion of the Golgi by BFA or GA reproduced the AJ/TJ disassembly and disruption of the paracellular barrier observed with αSNAP depletion (Figure 9). Loss of αSNAP did not simply disperse the Golgi ribbon, but also impaired the functional activity of this organelle that was revealed by defective glycosylation of plasma membrane adhesive proteins (Figure S3). We believe that effects of αSNAP knockdown are not limited to the Golgi architecture, but also involve alterations in the ER structure and functions. A diffuse patternless staining of canonical ER markers calnexin and calreticulin in control SK-CO15 cells did not allow direct examination of the effects of αSNAP depletion on the ER structure. However, the Golgi and the ER are known to be interconnected by the constant vesicle fluxes going either from the ER to Golgi (anterograde transport) or in the opposite direction (retrograde transport) [77], [78]. Because of this bidirectional vesicle flux, disruption of the Golgi should affect ER structure and *vice versa*. In support of this notion, we found that αSNAP knockdown dispersed the ERGIC (Figure 7), which can be explained by the decreased vesicle flow from the disordered and dysfunctional ER. It is expected that the observed alterations of the Golgi-ER dynamics can prevent assembly of junctional complexes at the plasma membrane via multiple mechanisms. These mechanisms may involve misfolding and abnormal posttranslational processing of AJ/TJ proteins and/or their defective sorting into vesicular carriers at the ER exit sites or in the trans-Golgi network.

Given our results suggesting that αSNAP depletion interrupts AJ/TJ biogenesis at the Golgi level, it is important to understand how loss of this membrane fusion protein disrupts Golgi architecture. Previous biochemical studies demonstrated a direct association of αSNAP with two distinct SNARE complexes involving either syntaxin-5 [43], [55] or syntaxin-18 [42], [45] that respectively control the anterograde or retrograde vesicle trafficking between the ER and Golgi [33]. Furthermore, αSNAP was shown to mediate fusion of ER-derived vesicles with Golgi membranes in a cell-free vesicle fusion reconstruction assay [56]. Therefore, one can predict that loss of αSNAP would inhibit disassembly/recycling of both the anterograde and the retrograde SNARE complexes at the ER and the Golgi. This would stop vesicle cycling between two organelles leading to their structural disintegration and functional deficiency. However, our results add another level of complexity to the mechanisms of αSNAP-dependent regulation of the Golgi by revealing expressional downregulation of Golgi-associated GEFs in αSNAP-depleted epithelial cells (Figure 9A). These GEFs, namely GBF1, BIG1 and BIG2 are known to activate ARF GTPases, which are essential for the assembly of coated vesicles in different regions of the Golgi and the ERGIC [79], [80]. Therefore, loss of αSNAP can simultaneously interfere with two critical trafficking steps at the Golgi such as ARF-dependent assembly of coated vesicles and SNARE-mediated vesicle fusion. Downregulation of Golgi GEFs can also contribute to the disruption of AJs and TJs in αSNAP-depleted cells. Although inhibition of all three Golgi GEFs by BFA was previously shown to induce AJ disassembly [81], we observed for the first time a unique role for GBF1 in maintaining normal AJ and TJ architecture and epithelial permeability (Figures 9, 10 & S4). These findings are consistent with reported non-redundant functions of the Golgi resident GEFs. For example, a specific pro-survival activity of GBF1, but not BIG1 or BIG2, was observed in human hepatoma cells [82]. This may reflect a unique role for GBF1 in vesicle delivery from the ERGIC to the cis-side of the Golgi, which is a key stage in Golgi ciscernae biogenesis [62].

Another important finding of this study is a role for αSNAP as a positive regulator of protein expression. Indeed, loss of αSNAP decreased expression of such functionally-diverse proteins as p120 catenin (Figure 3C), Golgi GEFs (Figure 9) and anti-apoptotic Bcl-2 (data not shown). At least for p120 catenin and Bcl-2, such expressional down-regulation cannot be explained by decreased mRNA transcription or increased protein degradation (data not shown), thereby suggesting defects in protein translation. Although mechanistic links between vesicle trafficking and protein translation have not been firmly established, the functional interplay between these two processes has been documented by previous studies. For example, enhancement of exocytic vesicle flux in cells overexpressing some SNARE and exocyst proteins resulted in the increased protein translation [83], [84]. On the other hand, BFA treatment that interrupts the ER-Golgi vesicle cycling was shown to inhibit protein synthesis [85]. Since vesicle trafficking can regulate several steps of polypeptide chain processing including ribosomal attachments to the ER membrane, translation initiation, and polypeptide translocation into the ER lumen [83], [84], it would be interesting to elucidate which steps of protein translation are impaired by αSNAP depletion.

In conclusion, our study reveals novel roles for a canonical membrane fusion protein αSNAP in the regulation of the structure and barrier function of epithelial apical junctions. αSNAP-dependent assembly of epithelial AJs and TJs appears to be mediated by previously unanticipated mechanisms that involve control of AJ protein expression and Golgi integrity and functions. Given the profound effects of αSNAP depletion on various cellular structures and processes, it would be important to investigate whether expression of this protein is affected in different diseases and if αSNAP dysfunction is involved in disassembly of epithelial junctions during mucosal inflammation and tumor metastasis.

## Materials and Methods

### Antibodies and other reagents

The following primary polyclonal (pAb) and monoclonal (mAb) antibodies were used to detect junctional, signaling and vesicle trafficking proteins: anti-αSNAP mAb (Abcam) anti-occludin, ZO-1, claudin-1, claudin-4, and JAM-A mAbs and pAbs (Invitrogen, Carlsbad, CA); anti-cleaved PARP, active caspase-3, active caspase-7 and GAPDH pAbs (Cell Signaling); anti-E-cadherin, β-catenin, GM-130 and NSF mAbs (BD Biosciences, San Jose, CA); anti-ERGIC53 mAb (Enzo Life Sciences, Plymouth Meetings, PA); anti-BIG1 and Giantin pAbs (Covance, Princeton, NJ); anti-β-catenin pAb (Sigma-Aldrich, St. Louis, MO); anti-α-catenin mAb (Epitomics, Burlingame, CA). Anti β1 integrin rat and rabbit mAbs were obtained from the University of Iowa Hybridoma Bank and Novus Biologicals (Littleton, CO) respectively. Polyclonal and monoclonal antibodies against p120 catenin were described previously [86], [87]. Anti-BIG2 pAb was provided by Dr. Martha Vaughan (National Heart, Lung and Blood Institute, NIH, Bethesda, MD). Alexa-488 or Alexa-568 dye conjugated donkey anti-rabbit and goat anti-mouse secondary antibodies, as well as Alexa-dye conjugated phalloidin were obtained from Invitrogen. Horseradish peroxidase-conjugated goat anti-rabbit and anti-mouse secondary antibodies were purchased from Jackson Immunoresearch Laboratories (West Grove, PA). Z-VAD-fmk was purchased from MP Biomedicals (Solon, OH). All other reagents were obtained from Sigma-Aldrich.

### Cell culture and calcium switch model

SK-CO15 (a gift from Dr. Enrique Rodriguez-Boulan, Weill Medical College of Cornell University, NY) and T84 (ATCC, Manassas, VI) human colonic epithelial cells (ATCC) were cultured as previously described [67], [88], [89], [90]. Cells were grown in standard T75 flasks, and for immunolabeling experiments, were plated on either collagen-coated, permeable polycarbonate filters 0.4 µm pore size (Costar, Cambridge, MA) or on collagen-coated coverslips. For biochemical experiments, the cells were cultured in 6-well plastic plates. To study AJ/TJ reassembly, confluent SK-CO15 cells were subjected to ‘calcium switch’ as previously described [67], [90].

### Immunofluorescence labeling and image analysis

Epithelial cell monolayers were fixed/permeabilized in 100% methanol for 20 min at −20°C. Fixed cells were blocked in HEPES-buffered Hanks balanced salt solution (HBSS^+^) containing 1% bovine serum albumin (blocking buffer) for 60 min at room temperature and incubated for another 60 min with primary antibodies diluted in the blocking buffer. Cells were then washed, incubated for 60 min with Alexa dye-conjugated secondary antibodies, rinsed with blocking buffer and mounted on slides with ProLong Antifade medium (Invitrogen). Immunofluorescently-labeled cell monolayers were examined using either an Olympus FluoView 1000 confocal microscope (Olympus America, Center Valley, PA) or a Zeiss LSM510 laser scanning confocal microscope (Zeiss Microimaging Inc., Thornwood, NY). The Alexa Fluor 488 and 568 signals were imaged sequentially in frame-interlace mode to eliminate cross talk between channels. The images were processed using either the Olympus FV10-ASW 2.0 Viewer, or Zeiss LSM5 Image Browser software and Adobe Photoshop. Images shown are representative of at least 3 experiments, with multiple images taken per slide. Quantification of Golgi disruption was performed by visually examining Golgi morphology in ten 100× fields per each experimental group (∼43 cells per field), that were acquired in three independent experiments.

### Immunoblotting

Cells were homogenized in RIPA lysis buffer (20 mM Tris, 50 mM NaCl, 2 mM EDTA, 2 mM EGTA, 1% sodium deoxycholate, 1% Triton X-100 (TX-100), and 0.1% SDS, pH 7.4), containing a protease inhibitor cocktail (1∶100, Sigma) and phosphatase inhibitor cocktails 1 and 2 (both at 1∶200, Sigma). Lysates were cleared by centrifugation (20 min at 14,000× g), diluted with 2xSDS sample buffer and boiled. SDS-polyacrylamide gel electrophoresis and immunoblotting were conducted by standard protocols with an equal amount of total protein (10 or 20 µg) per lane. Protein expression was quantified by densitometry of three immunoblot images, each representing an independent experiment, with a Kodak Image Station 2000R and Kodak Molecular Imaging software V 4.0 (Eastman Kodak, Rochester, NY). Data are presented as normalized values assuming the expression levels in control siRNA-treated groups were at 100%. Statistical analyses were performed with row densitometric data using the Microsoft Excel program.

### RNA interference

siRNA-mediated knockdown of αSNAP, NSF, p120 catenin, GBF1, BIG1 and BIG2 was carried out as previously described [67], [68], [90]. Individual siRNA duplexes (Dharmacon, Lafayette, CO) were used to downregulate αSNAP expression, whereas knockdown of other targets was performed by using specific siRNA SmartPools (Dharmacon). A non-coding siRNA duplex-2 or cyclophilin B SmartPool (Dharmacon) was used as a control. SK-CO15 cells were transfected using the DharmaFect 1 reagent (Dharmacon) in Opti-MEM I medium (Invitrogen) according to the manufacturer's protocol with a final siRNA concentration of 50 nM. Cells were utilized in experiments on days 3 and 4 post-transfection.

### Preparation of αSNAP- and p120-overexpressing epithelial cells

For the rescue experiments, the coding sequence of aSNAP (gift from Dr. Reinhard Jahn, Max Planck Institute for Biophysical Chemistry, Gottingen, Germany) was cloned in pLXSN retroviral vector (Clontech) as a BamH1, EcoR1 fragment. The bovine αSNAP transcript has 4 nucleotide mismatches in the corresponding sequences and is not targeted by the siRNA duplexes 1 and 2 used to deplete human αSNAP in our experiments. For retroviral particle production, ProPak-A cells (ATCC) were transfected with either empty control vector or bovine αSNAP-containing vector using a Trans-IT 293 transfection reagent (MirusBio, Madison, WI). p120 catenin isoforms were cloned into the LZRS retroviral system as described at length in a previous publication [91]. For production of retroviruses, Phoenix 293 cells were transfected by calcium phosphate with LZRS-IRES-neo empty retroviral vector or vectors bearing mp120-1A or mp120-3A and transfected cells were selected with 1 µg/ml G418. In both cases in order to harvest the viruses, infected cells growing at 75% confluence were incubated for 16 h at 37°C. Collected media was passed through a 0.45-µm syringe filter and stored at −80°C. SK-CO15 cells plated at 30–40% confluence were exposed overnight to viruses preincubated with 4 µg/ml polybrene. Stable cell lines expressing either bovine αSNAP, or p120 catenin isoforms along with their appropriate controls (SK-neo) were selected with 1.2 µg/ml G418.

### Epithelial barrier permeability measurements

TEER was measured with an EVOMX voltohmmeter (World Precision Instruments, Sarasota, FL). The resistance of cell-free collagen-coated filters was subtracted from each experimental point.

### Statistics

Numerical values from individual experiments were pooled and expressed as mean ± standard error of the mean (S.E.) throughout. Obtained numbers were compared by two-tailed Student's *t*-test, with statistical significance assumed at p<0.05.

## Supporting Information

Figure S1 **Junctional disassembly in αSNAP-depleted cells is independent of cell death.** To exclude a possible role of two major cell death pathways in junction disassembly, SK-CO15 cells were transfected with either control or αSNAP-specific siRNA (duplex 1), and one day later, were exposed to either vehicle or a combination of apoptosis inhibitor Z-VAD-fmk (50 µM) and necroptosis inhibitor necrostatin (Nst)-1 for 72 h. A dual inhibition of apoptosis and necroptosis did not affect structure of normal AJs and TJs (arrows) but failed to prevent junctional disassembly in αSNAP-depleted SK-CO15 cells (arrowheads). Scale bar, 20 µm.
(TIF)

Figure S2 **Overexpression of p120 catenin does not prevent junctional disassembly in αSNAP-depleted cells.** (**A**) Immunoblotting analysis shows preserved p120 catenin level in p120-3 overexpressing SK-CO15 cells after αSNAP depletion. However, such p120-3 overexpression does not prevent disruption of the paracellular barrier (**B**) and disassembly of AJs and TJs (**C, arrows**) in αSNAP-depleted epithelial cells; ^#^p<0.01 compared to the control siRNA-treated group (n = 3). Scale bar, 20 µm.
(TIF)

Figure S3 **Downregulation of αSNAP impairs glycosylation and plasma membrane delivery of β1 integrin.** (**A**) SK-CO15 cells were transfected with either control or two different αSNAP duplexes (D1 and D2). Immunoblotting analysis shows the increased intensity of the lower band of β1 integrin in αSNAP-depleted cells that corresponds to its nonglycosylated form. (**B**) Immunofluorescence labeling shows plasma membrane localization of β1 integrin in control cells (arrows) and intracellular accumulation of this protein on day 4 of αSNAP knockdown (arrowheads). The lower panel of images presenting a dual immunolabeling with rat β1 integrin antibody and control rabbit IgG, confirms the specificity of β1 integrin staining. Scale bar, 20 µm.
(TIF)

Figure S4 **Downregulation of BIG1 and BIG2 expression does not affect the integrity of epithelial apical junctions.** Immunofluorescence labeling shows normal architecture of β-catenin-based AJs and occludin/ZO-1-based TJs in either BIG1 or BIG2-depleted SK-CO15 cells on day 4 post-transfection. Scale bar, 20 µm.
(TIF)
