# Supplementary figures and images for: A Membrane Fusion Protein αSNAP Is a Novel Regulator of Epithelial Apical Junctions
Source: PLoS One. 2012 Apr 2;7(4):e34320. doi: 10.1371/journal.pone.0034320 (PMC3317505; doi:10.1371/journal.pone.0034320)

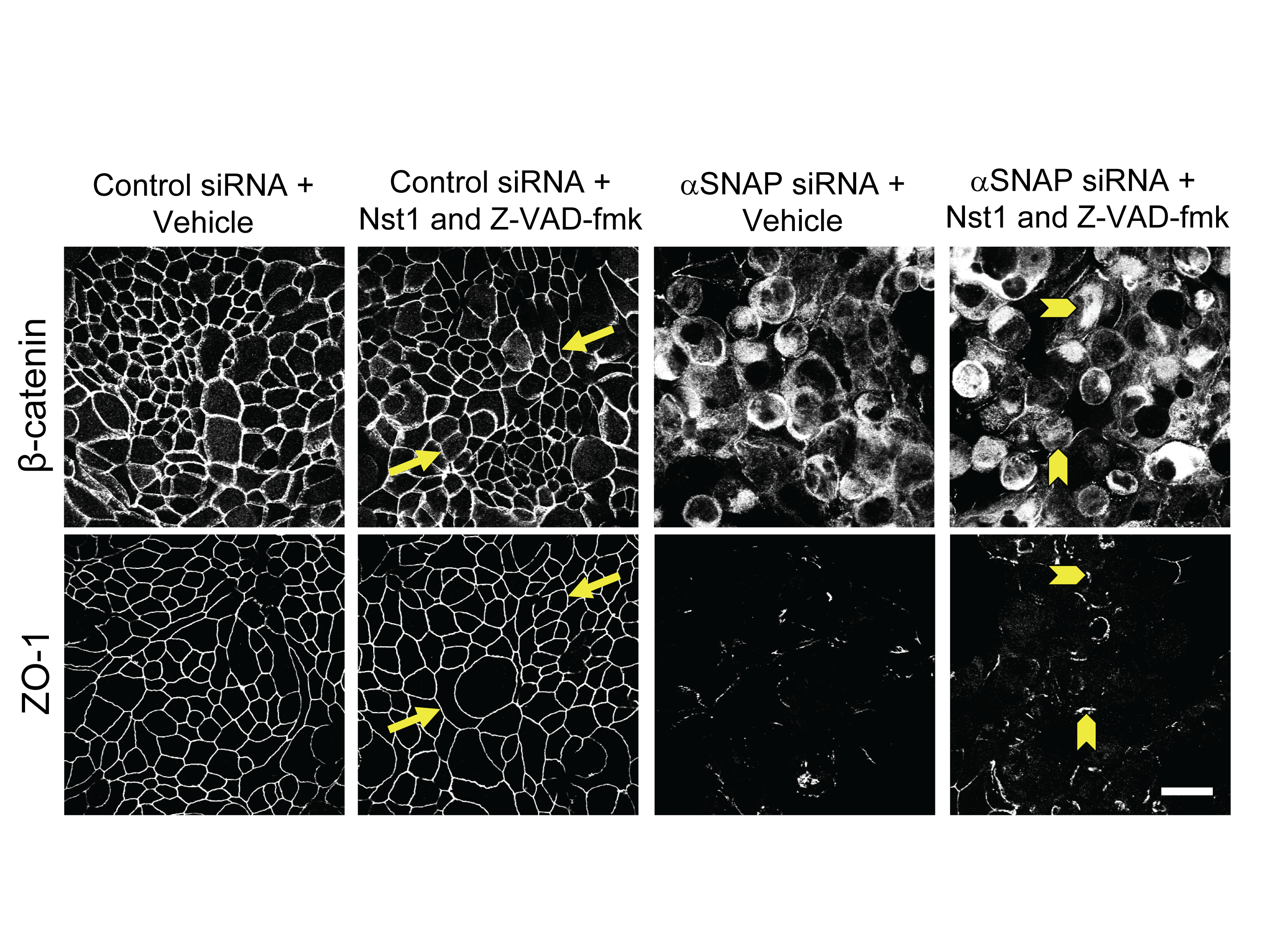

Supplement: Figure S1 — Junctional disassembly in αSNAP-depleted cells is independent of cell death. To exclude a possible role of two major cell death pathways in junction disassembly, SK-CO15 cells were transfected with either control or αSNAP-specific siRNA (duplex 1), and one day later, were exposed to either vehicle or a combination of apoptosis inhibitor Z-VAD-fmk (50 µM) and necroptosis inhibitor necrostatin (Nst)-1 for 72 h. A dual inhibition of apoptosis and necroptosis did not affect structure of normal AJs and TJs (arrows) but failed to prevent junctional disassembly in αSNAP-depleted SK-CO15 cells (arrowheads). Scale bar, 20 µm. (TIF) [file pone.0034320.s001.tif]

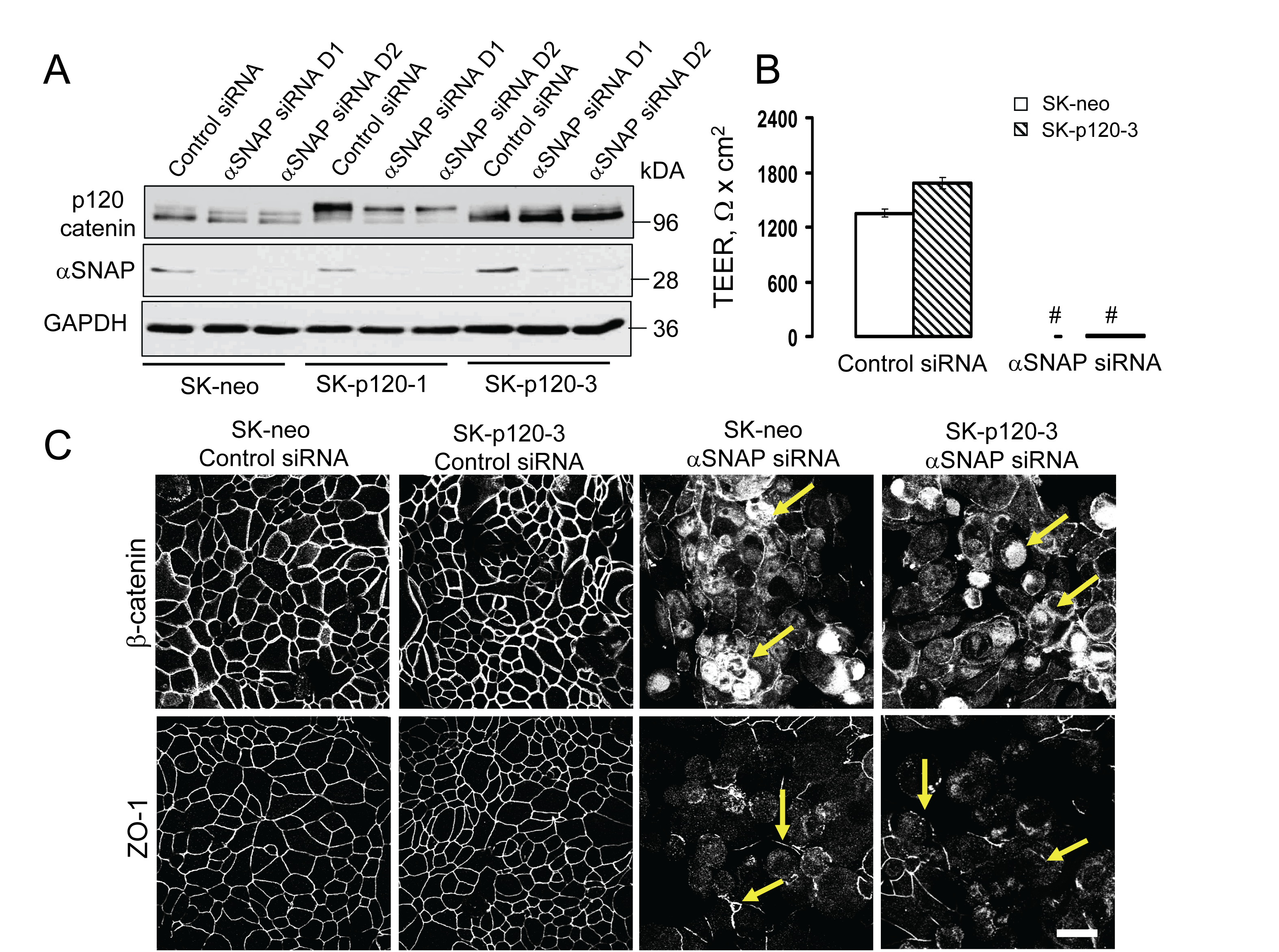

Supplement: Figure S2 — Overexpression of p120 catenin does not prevent junctional disassembly in αSNAP-depleted cells. (A) Immunoblotting analysis shows preserved p120 catenin level in p120-3 overexpressing SK-CO15 cells after αSNAP depletion. However, such p120-3 overexpression does not prevent disruption of the paracellular barrier (B) and disassembly of AJs and TJs (C, arrows) in αSNAP-depleted epithelial cells; #p<0.01 compared to the control siRNA-treated group (n = 3). Scale bar, 20 µm. (TIF) [file pone.0034320.s002.tif]

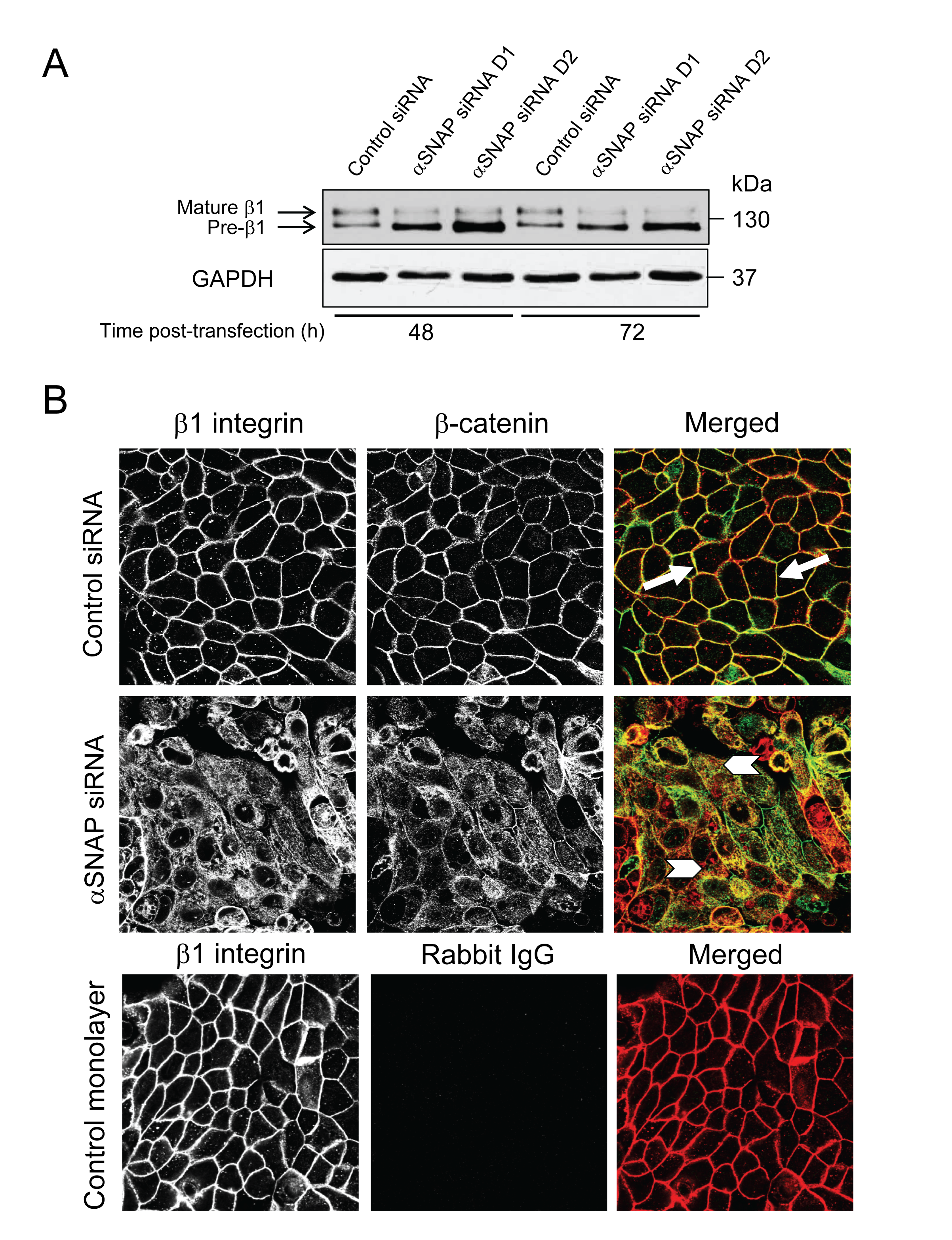

Supplement: Figure S3 — Downregulation of αSNAP impairs glycosylation and plasma membrane delivery of β1 integrin. (A) SK-CO15 cells were transfected with either control or two different αSNAP duplexes (D1 and D2). Immunoblotting analysis shows the increased intensity of the lower band of β1 integrin in αSNAP-depleted cells that corresponds to its nonglycosylated form. (B) Immunofluorescence labeling shows plasma membrane localization of β1 integrin in control cells (arrows) and intracellular accumulation of this protein on day 4 of αSNAP knockdown (arrowheads). The lower panel of images presenting a dual immunolabeling with rat β1 integrin antibody and control rabbit IgG, confirms the specificity of β1 integrin staining. Scale bar, 20 µm. (TIF) [file pone.0034320.s003.tif]

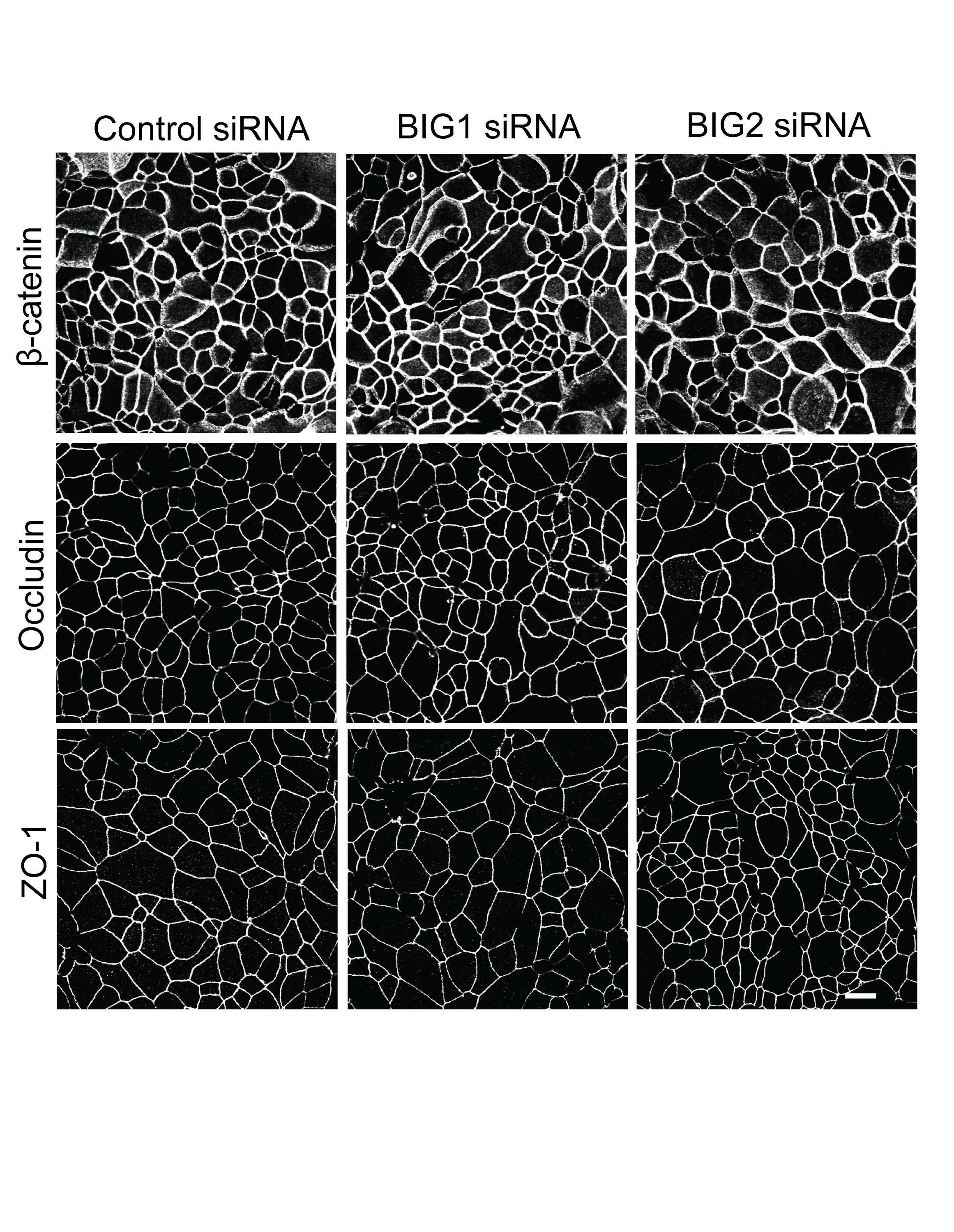

Supplement: Figure S4 — Downregulation of BIG1 and BIG2 expression does not affect the integrity of epithelial apical junctions. Immunofluorescence labeling shows normal architecture of β-catenin-based AJs and occludin/ZO-1-based TJs in either BIG1 or BIG2-depleted SK-CO15 cells on day 4 post-transfection. Scale bar, 20 µm. (TIF) [file pone.0034320.s004.tif]
